# A Hybrid Scheme for Disaster-Monitoring Applications in Wireless Sensor Networks

**DOI:** 10.3390/s23115068

**Published:** 2023-05-25

**Authors:** Danqi Chen, Yanxia Zhang, Guoli Pang, Fangping Gao, Li Duan

**Affiliations:** 1 School of Information Engineering, Institute of Disaster Prevention, Sanhe 065201, China; zhangyanxia@cidp.edu.cn (Y.Z.); pangguoli@cidp.edu.cn (G.P.); gaofangping@cidp.edu.cn (F.G.); 2 College of Electronic Science and Control Engineering, Institute of Disaster Prevention, Sanhe 065201, China; duanli@cidp.edu.cn

**Keywords:** hybrid scheme, frame delay, throughput, lifetime, wireless sensor networks, disaster-monitoring applications

## Abstract

Disaster monitoring is a primary task for wireless sensor networks. Systems for the rapid reporting of earthquake information are a crucial aspect of disaster monitoring. Furthermore, during emergency rescue after a large earthquake, wireless sensor networks can provide pictures and sound information to save lives. Therefore, when accompanied by multimedia data flow, the alert and seismic data sent by the seismic monitoring nodes must be sufficiently fast. We present herein the architecture of a collaborative disaster-monitoring system that can obtain seismic data in a highly energy-efficient manner. In this paper, a hybrid superior node token ring MAC scheme is proposed for disaster monitoring in wireless sensor networks. This scheme consists of set-up and steady-state stages. A clustering approach was proposed for heterogeneous networks during the set-up stage. The proposed MAC operates in the duty cycle mode at the steady-state stage and is based on the virtual token ring of ordinary nodes, the polling all the superior nodes in one period, and alert transmissions with a low-power listening and shortened preamble approach during the sleep state. The proposed scheme can simultaneously satisfy the requirements of three types of data in disaster-monitoring applications. Based on embedded Markov chains, a model of the proposed MAC was developed and the mean queue length, mean cycle time, and mean upper bound of the frame delay were obtained. Using simulations under various conditions, the clustering approach performed better than the pLEACH approach, and the theoretical results of the proposed MAC were verified. We found that alerts and superior data have outstanding delay and throughput performances even under heavy traffic intensity, and the proposed MAC can provide a data rate of several hundred kb/s for superior and ordinary data. Considering all three types of data, the frame delay performances of the proposed MAC are better than those of the WirelessHART and DRX schemes, and the alert data of the proposed MAC have a maximum frame delay of 15 ms. These satisfy the application requirements of disaster monitoring.

## 1. Introduction

Wireless sensor networks (WSNs) are applied far and wide for environmental monitoring, smart homes, intelligent transportation, health monitoring, and numerous other fields [1,2]. Over the last ten years, substantial progress has been made in wireless communication and low-power electronics, and small sensors have considerably promoted the development and application of WSNs. Generally, the sensor nodes of WSNs consist of automated sensing, processing, wireless communication, and power supply units. The information gathered from sensor nodes is transmitted to a base station for further processing and analyses [3]. With the development of MEMS technology, multimedia information such as sounds, pictures, and videos can be sensed by sensor nodes. Multimedia information is paramount for several WSNs applications. However, multimedia sensing is challenging, considering the small sensor nodes that have limited processing and power supply. Meanwhile, it is difficult for small sensor nodes to perform data-sensing and network transmission functions and satisfy the requirements of the quality of service (QoS) for multimedia communication in WSNs. Owing to technological progress and the diverse sensing requirements of WSN applications, application-specific QoS provisioning techniques have recently been confirmed as crucial research areas for WSNs [4,5].

### 1.1. Motivation

Disaster monitoring is critical for environmental monitoring. To protect against and mitigate the damage caused by earthquake disasters, three tasks should be performed: earthquake prediction, emergency management of earthquake hazards, and recovery from earthquakes [6]. Earthquake prediction is a scientific challenge worldwide and is still in the research and exploration stages. Earthquake early warning (EEW) is a feasible strategy to mitigate the effects of seismic hazards [7]. The essential task is to save lives during the emergency management of earthquake hazards. When large earthquakes occur, buildings and facilities are destroyed, and communication is intermittent. Under these circumstances, if an early warning of a violent earthquake is not provided, severe damage can occur. By taking advantage of electromagnetic waves that spread faster than seismic waves, an early warning can be provided when an earthquake occurs. This may not only immediately stop high-speed trains, nuclear power stations, and electric elevators in high-rise buildings, but may also provide people with precious time of more than 10 s to save lives, especially the lives of those who are approximately 60 km from the epicenter [8]. The seismic station is the basic setting of seismic monitoring. Seismic stations use geophones to detect earthquakes. Geophones are an electromechanical conversion device used to convert the seismic waves transmitted to the ground into electrical signals. They are the key piece of equipment involved in field data collection by seismic stations [9]. WSNs are a noteworthy method for implementing EEW. WSNs act as part of disaster-monitoring systems, and they have a considerable advantage over other networks because they do not depend on any other infrastructure, such as NB-IoT and 5G wireless networks [10]. When a large earthquake occurs, mobile communication base stations and fiber-optic networks are simultaneously destroyed. In this situation, WSNs can help disaster-monitoring systems to keep working. However, wireless sensor networks must achieve real-time transmission to perform this task. A system for EEW is a vital aspect of disaster monitoring because it allows people to survive earthquakes. Normally, an onsite earthquake early warning system measures the peak displacement amplitude and predominant period of the P-wave for 3–4 s to avoid frequent false alarms. Therefore, alerts sent by seismic monitoring nodes must be transmitted within tens of milliseconds in a wireless sensor network [11].

However, a violent earthquake can severely damage buildings and facilities and interrupt transportation systems and communication networks [12]. Maintaining contact with people in the post-earthquake regions is difficult. When emergency rescue is performed, landslides, mudrock flows, and aftershocks can lead to further casualties and damage. To mitigate this, real-time and accurate seismic data must be obtained by deploying robust sensor nodes in disaster areas for uninterruptible monitoring. To transmit post-earthquake disaster data, a rapid reporting system based on wireless sensor networks is an efficient strategy. After an earthquake, the nodes of the wireless sensor networks can be dropped onto the disaster area using unmanned aerial vehicles. In particular, when current information networks are interrupted, we can send important, timely information by taking advantage of these nodes. It is paramount that seismic monitoring nodes can transmit epicenter vibration information so that aftershock alerts from the seismic monitoring nodes in the system can be transmitted quickly. Moreover, the goal is to obtain information about earthquake areas, and multimedia information about disaster situations is extremely important [13].

In another disaster-monitoring scenario, volcanic research and monitoring are vital for responding to volcanic eruption disasters [14]. This monitoring system must also satisfy the requirements of short delay in alert messages and high throughput of multimedia information for volcano disaster monitoring.

### 1.2. Problem Statement

Satisfying QoS requirements is the most important issue in the disaster-monitoring application domain, and the medium access control (MAC) layer plays a critical role. However, WSNs exhibit dynamic network environments, heterogeneous network traffic, and resource-constrained nodes. Disaster monitoring involves three main metrics: delay, throughput, and reliability. Alert messages should be transmitted as quickly as possible, and this delay plays an important role. The MAC layer can obtain minimum delay by minimizing queuing and transmission delays. Sufficient throughput is required for multimedia transmission in WSNs. Because wireless communication is based on a shared medium, all co-instantaneous transmissions in the coverage of wireless signals interfere with each other, and this interference causes frame loss. This affects transmission delay and network throughput. The reliability of disaster monitoring is vital, and WSNs must provide reliable transmission, even in abnormal situations.

In general, three types of data are available for disaster-monitoring applications. One is ordinary data, such as sound, pictures, or video data, which require high throughput, such as data rates of several hundred kb/s for 480p H.265 video streaming [15], and can endure some degree of frame loss. The second type of data, seismic data, simultaneously require a high throughput of several kb/s and a low delay [11]. The third type is alert data, which demand a low delay of tens of milliseconds and high reliability. In a disaster-monitoring system with WSNs, the MAC layer of the WSNs must simultaneously satisfy the requirements of the above three types of data, which is a difficult and vital issue.

### 1.3. Contributions

In this study, we propose a hybrid scheme of a MAC layer and a collaborative disaster-monitoring system for disaster-monitoring applications. The key contributions of this study are as follows.

- A hybrid superior node token ring MAC (HT-MAC) scheme is proposed, which is based on the virtual token ring of ordinary nodes, the polling of all superior nodes in one period, and alert transmissions with a low-power listening (LPL) and shortened preamble approach during the sleep state. A low-power clustering method for heterogeneous WSNs is proposed in order to increase the lifetime of disaster-monitoring systems;
- Based on embedded Markov chains, a model of the HT-MAC scheme was developed, and the mean queue length, mean cycle time, and mean upper bound of the frame delay were obtained;
- Using the HT-MAC, WirelessHART, and DRX simulation models, simulations were performed under various conditions, and the delay specifications for the three types of data were explored. The theoretical results were verified through simulations. Based on the simulation results, this HT-MAC scheme satisfies the delay and throughput requirements for the three types of data in disaster-monitoring applications, and can enhance the effective lifetime of heterogeneous networks with the proposed clustering algorithm;
- An architecture for a collaborative disaster-monitoring system is proposed, and a method for obtaining seismic data using a highly energy-efficient approach is presented.

### 1.4. Paper Organization

The remainder of this paper is organized as follows: Section 2 discusses the related literature and presents the chief objective for disaster-monitoring applications based on wireless sensor networks. In Section 3, we describe the design requirements for the collaborative disaster-monitoring system and explain its architecture. We then analyze how to acquire seismic data in a highly energy-efficient manner. Section 4 presents the proposed HT-MAC scheme and analyzes the clustering, data transmission, and abnormity-handling operations. In Section 5, based on the embedded Markov chain method, the formulas for the mean queue length, mean cycle time, and mean upper bound of the frame delay are derived. Section 6 presents the simulations and performance tests of the HT-MAC scheme, and the results are analyzed. Finally, Section 7 presents the conclusions and future work of this study.

## 2. Related Work

Wireless sensor networks have been extensively applied in many areas. They do not depend on any supporting infrastructure, and therefore have special advantages over other networks in terms of disaster monitoring. The MAC layer plays a vital role in disaster-monitoring systems. Thus far, various relevant MAC protocols for WSNs have been developed, mostly based on contention-based, schedule-based, hybrid, or multiple wireless interface mechanisms, as shown in Table 1. A comparison of the relevant MAC protocols is presented in Table 2.

### 2.1. Contention-Based MAC

The random multiple access method with collision avoidance is a fundamental contention-based mechanism for WSNs, and many protocols have been presented and studied, such as the S-MAC, B-MAC, and PMAC protocols [16,17,18]. To achieve energy efficiency, S-MAC reduces idle listening using a periodic duty cycle scheme [16]. Within an active period, the sensor nodes of the S-MAC transmit messages according to the IEEE 802.11 standard. The S-MAC protocol uses the CSMA/CA mechanism and request-to-send (RTS)/ clear-to-send (CTS) to maintain collision avoidance. S-MAC undergoes a high delay because packets are queued at a node until the next active period.

To mitigate uncertain delays between source and sink nodes, the RT-MAC protocol overcomes the false blocking problem when RTS/CTS packets are exchanged, and maximizes spatial channel reuse [19]. However, the interference caused by multiple communication streams still affects the RT-MAC protocol. To obtain high throughput and low delay performance while considering the data input rate, the MaxMAC protocol takes advantage of an added wake-up scheme [20]. However, MaxMAC cannot respond accurately to traffic changes. In the ENCO protocol, optimization of the contention window size approximation for the number of contending nodes is used to reduce the contention delay [21]. ENCO requires extra computational overhead to optimize contention window sizes. Based on the IEEE 802.15.4 unslotted CSMA/CA scheme, QoS-MAC sets different priorities with differential services for data traffic to obtain QoS support, and this protocol is applied to the vital distribution monitoring of smart grids [22]. However, the QoS-MAC suffers from severe collisions when the traffic data rate increases. Based on data prioritization and delay estimation methods, DRX was designed for smart grid applications, and the MAC layer parameters responded to the delay requirements of the application and network conditions [23]. In DRX, the packet delivery ratio is reduced when the number of nodes increases, because the number of collisions increases.

PW-MAC is an energy-efficient MAC protocol based on an asynchronous duty cycle [24]. The sender of the PW-MAC wakes up and turns on its radio immediately before the intended receiver wakes up. The sensor nodes in PW-MAC compute their wake-up times using their pseudorandom wake-up schedule generator, which reduces radio collisions caused by multiple concurrent traffic flows. However, PW-MAC addresses the real-time bursting of traffic. CSMA/WSD is a variant of CSMA that implements weak signal detection to mitigate energy consumption [25]. Although this approach allows the neighbor node to detect a weak signal and gain more throughput by performing a loss diagnosis, CSMA/WSD requires a dense deployment of sensor nodes.

### 2.2. Schedule-Based MAC

The random multiple access protocol can efficiently utilize the wireless channel compared to fixed-assigned multiple access protocols. However, when the traffic intensity increases, frame collisions of random multiple access protocols increase. Consequently, the throughput decreases and the delay increases. For real-time applications, an approach based on scheduling may avoid frame collisions and is considered the primary approach. Time-division multiple access (TDMA) is a basic scheduling protocol. In the TDMA protocol, time slot allocation requires a global view and extensive computation. Several TDMA protocols such as the BMA protocol take advantage of the characteristics of cluster networks, ease of management and maintenance, and rapid response to system changes [26]. In the BMA protocol, the nodes are selected as cluster heads according to the residual energy, and each time frame is divided into three fixed parts, a contention period, a data transmission period, and an idle period, which cannot adapt to changes in network traffic, and reduce channel utilization.

Considering the load density changes in communication, TDMA may dynamically allocate slots; therefore, this approach can substantially promote throughput and delay performance [27]. The TRAMA protocol ensures that nodes use the slot according to the actual traffic and that nodes without communication tasks sleep, thereby reducing the energy consumption caused by conflict and idle listening [28]. In TRAMA, random and scheduled access alternately increase end-to-end delay, and idle listening wastes energy. The TDMA-W protocol [29] improves TRAMA using a fixed time slot to send or receive data. TDMA-W utilizes a distributed algorithm similar to graph coloring to assign slots to all the nodes. The sleeping delay problem is a major defect in TDMA-W.

For environmental monitoring applications, a balance is required between the delay and the energy consumption of WSNs. ArDez takes advantage of a pseudorandom number scheme to establish a channel, and is a TDMA protocol based on rendezvous periods [30]. The ArDeZ protocol requires no scheduling negotiation or strict time-slot boundaries, and the collision probability is very low, achieving a balance between delay and energy consumption. However, ArDez cannot support broadcasting well, and the nodes in the main path consume too much energy. Considering the data-forwarding interruption problem, DMAC adopts a staggered wake-up scheduling mechanism in the data-gathering tree [31]. DMAC supports packets that are continuously forwarded, and is conducive to the timely reporting of perceptual data. DMAC is unfavorable for interest queries and instruction transmissions.

### 2.3. Hybrid MAC

Hybrid mechanisms typically support additional features. Based on the advantages of both the CSMA and TDMA approaches, CSMA-STDMA is a self-organized MAC protocol, based on hybrid mechanisms, that provides reliable and real-time wireless network communications [32]. CSMA-STDMA utilizes the CSMA mechanism to provide synchronization among nodes, and then transmits data in a self-organized TDMA manner. CSMA-STDMA suffers from frequent initialization and energy consumption during idle listening.

To meet different packet requirements, the E-hybrid protocol operates in the contention period and priority schedule period based on CSMA and TDMA, respectively [33]. When the data traffic is low, the E-hybrid protocol increases the contention access period. Otherwise, E-hybrid extends the reserved slots to the scheduling mode in high-traffic conditions, or according to urgent data, implements an emergent contention-free period at the beginning of the super frame. According to industrial process-controlling applications, WirelessHART is the preferred wireless communication standard based on the IEEE 802.15.4 protocol [34]. Utilizing the advantages of CSMA and TDMA, WirelessHART is a hybrid scheme for strict real-time and high-security industrial applications. However, it is difficult for the two protocols described above to deal with rapid changes in traffic, such as in disaster-monitoring applications.

One of the goals of hybrid schemes is to satisfy throughput and delay requirements. Z-MAC is a hybrid scheme of CSMA and TDMA [35]. CSMA channel access under low traffic conditions improves channel utilization and reduces latency, and the TDMA channel mode under high traffic conditions can reduce collisions and interference. In Z-MAC, centralized scheduling allocation can only slot for nodes at the initial stage of the protocol and cannot be rerun periodically. Based on the control information between the nodes, a token cycle scheduling method called TCS was proposed to improve the throughput and delay performance of the TDMA approach [36]. This scheme is adapted for low-delay applications; however, it requires an additional control channel.

### 2.4. Multiple Wireless Interfaces MAC

In general, a sensor node has only one wireless communication interface. If sensor nodes have multiple wireless communication interfaces, this may naturally improve the throughput and delay performance. MMAC-HR has two wireless interfaces; one is used as the control channel to exchange control information, and the other shifts dynamically among the data channels [37]. Therefore, MMAC-HR resolves the multiple-channel exposed terminal problem and can enhance the throughput and delay performance. The DSP is a MAC protocol based on multiple-interface nodes and fast- and slow-hopping approaches with multiple-channel access [38]. To avoid the busy receiver problem, the DSP protocol utilizes a multiple rendezvous approach to reduce the delay, because the slow-hopping interface always follows the same hopping sequence. Multiple wireless interface MAC protocols require hardware support and consume more energy.

### 2.5. Clustering Methods

The nodes of wireless sensor networks are equipped with limited battery power, and perform the task of real-time surveillance in the disaster-monitoring system. Node-clustering is a promising method for maximizing their lifetime and satisfying the delay requirement. Numerous clustering methods have been investigated for homogenous sensor networks, such as LEACH [39] and DWEHC [40]. E-CERP clusters sensor nodes based on an adaptive sailfish optimization algorithm with K-medoids, and can attain a high network lifetime [41]. Heterogeneous clustering methods support networks with sensor nodes of different abilities. A stable election protocol (SEP) has been proposed for the heterogeneous routing protocol [42]. In this protocol, nodes are classified into normal and advanced nodes according to the initial energy and equipment; thus, different types of nodes gain different probabilities of becoming the cluster head. SEP does not use the residual energy of nodes to balance the energy consumption. Based on SEP, DEEC is a combination clustering method of centralization and distribution for heterogeneous networks [43]. DEEC selects the cluster head based on the initial and residual energy level of the nodes. To regulate the energy consumption of the adaptive approach, DEEC utilizes the average energy of the network to select the cluster head for attaining the long lifetime of networks. However, the base station of DEEC owns global knowledge of the networks, and we have not taken full advantage of this aspect. BIDRP is a dynamic routing protocol based on centralized clustering algorithms for heterogeneous wireless sensor networks [44]. In this protocol, nodes with more energy, computation capability, and location awareness are selected as the cluster head. In a cluster, transmission between the cluster head and sensing nodes occurs in a single hop, whereas transmission between different cluster heads occurs in multiple hops. However, the transmission of multiple hops is hardly to support real-time applications.

In this study, we propose a hybrid superior node token-ring MAC scheme, HT-MAC, for disaster-monitoring applications, such as the rapid reporting of earthquake information. Alerts and seismic information play important roles in disaster monitoring. Therefore, the delay and throughput of WSNs for alerts and seismic data must be guaranteed. HT-MAC operates in the duty cycle mode. Owing to the relatively short active state of the sensor nodes, low-traffic alert data are transmitted with a low-power listening approach during the sleep state to satisfy the delay requirements. By setting up a polling approach for superior nodes, this scheme can support low-delay transmissions in various traffic scenarios and provide flexible adaptation to traffic changes. This satisfies the delay and throughput requirements for disaster monitoring. Reliability is an important aspect of disaster monitoring, and WSNs should provide reliable transmission even in abnormal situations.

## 3. Collaborative Disaster-Monitoring System

Based on WSNs, earthquake information can be collected and delivered to seismic areas. With rapid advances in communication, embedded computing, and sensing techniques, various WSNs have been developed. WSNs can perform collaborative real-time monitoring, sensing, and gathering of data in various environments or surveillance objects. These data are processed to obtain accurate information that is finally delivered to the user. WSNs can obtain massive, detailed, and reliable physical world information at any time or place. However, for a disaster-monitoring system, the primary goal is to transmit alerts and superior information as quickly as possible, even when the network is under a heavy load. The limited computing power, communication ability, and battery energy of the heterogeneous nodes for wireless sensor networks must be considered.

**Figure 1:**
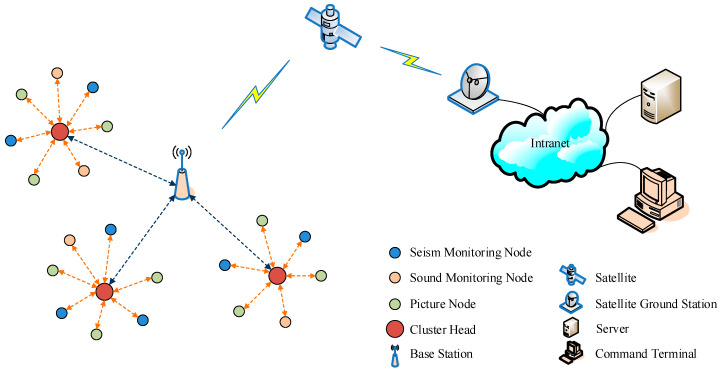
Network topology diagram of the collaborative disaster-monitoring system.

A network topology diagram of the collaborative disaster-monitoring system is shown in Figure 1. The system is a three-layered architecture of the Internet of Things. It consists of three parts: the wireless sensor networks as the perception layer, the satellite communication part as the communication layer, and the service center as the application layer. The wireless sensor network contains three types of sensor node: the seismic, picture, and sound nodes. Each node has two wireless communication interfaces. First, all sensor nodes are divided into clusters, and the node with the highest relative energy in a cluster is selected as the cluster head. Subsequently, in-cluster communication is performed using a hybrid superior node token ring scheme with one communication interface. Sensor nodes transmit data to the cluster head via a one-hop transmission. Communication between the cluster heads and the base station follows the TDMA mode with another communication interface. Seismic monitoring nodes gather seismic information using a seismometer. Picture-monitoring nodes capture pictures of a scene, and sound-monitoring nodes capture sound signals. These nodes then transmit information to the base station. Different types of nodes are equipped with batteries of different capacities. The base station contains a satellite transceiver and a battery rechargeable with solar energy. The satellite communication part is responsible for the long-distance transmission of information. It does not rely on the ground-based infrastructure. The base station delivers the collected information to the service center via satellites, satellite ground stations, and internal networks. The server of the service center can process all kinds of information both in time and effectively. Thus, based on the satellite link, disaster information can be obtained and processed even when the ground-based communication infrastructure in the earthquake zone is destroyed.

Seismic data can be treated as continuous variables in disaster-monitoring applications in WSNs. Continuous values are collected from spot observations; hence, each piece of data collected is important and indispensable. However, this may result in an excessive burden on the small nodes of the WSNs. Benefiting from advances in information technology, the method of compressed sensing for WSNs is effective for reducing this burden. Assuming that the distribution of data is sparse in a certain domain, this type of data collection with sensor nodes can be compressed using the compressed sensing method. In a collaborative disaster-monitoring system based on the compressed sensing method, seismic data can be obtained in an energy-efficient manner.

This system provides real-time and efficient ground vibration data, events, and alerts through satellite links. First, the seismic monitoring nodes perform compressive sampling of seismic variables and then deal with the compressive sampling data, alert them to network packets, and send them to the base station. The base station has a higher computing power and a larger solar energy supply than the sensor nodes. Therefore, at the base station, the system reconstructs seismic data and detects seismic events in accordance with our belief propagation fusion method [45]. This system transmits earthquake data, events and alerts to the server through a satellite link. The task of the disaster-monitoring system is to maintain the surveillance of earthquake activities, and most importantly, transmit alerts for dangerous vibrations as quickly as possible. After earthquake alerts are received and broadcast by the system server, people around the earthquake epicenter can take prompt action to avoid and mitigate damage.

## 4. Scheme Description

In disaster-monitoring systems, the MAC protocol plays the most important role in terms of delay, throughput, and reliability. The distributed polling scheme is an important type of the MAC protocol. In WSNs, polling involves sending a wireless query to each node and waiting for a unique response from the addressed node. This method supports real-time and high-throughput applications, and can automatically adapt to changes in traffic. For a distributed polling scheme in WSNs, a particular token frame is assigned to allocate wireless resources, and this token is transmitted along a virtual logical ring. Although the distributed polling scheme requires nodes of a cluster to be within a one-hop range, it provides high real-time and abnormity-handling power. Abnormity handling guarantees that the failure of any node cannot invalidate the entire network using a distributed polling scheme. This can significantly improve the reliability of WSNs in disaster-monitoring systems.

**Figure 2:**
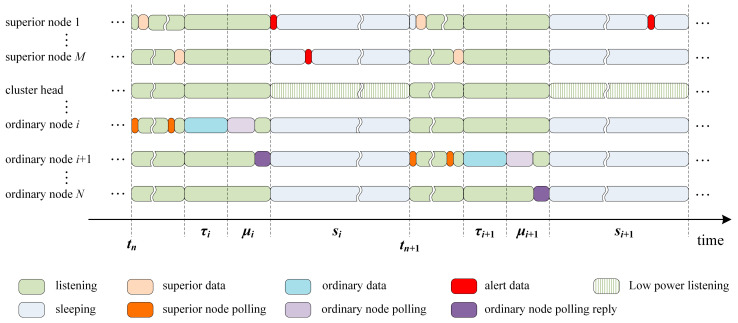
Timeline of the HT-MAC scheme at the steady-state stage.

The nodes of WSNs are divided into two types for disaster-monitoring applications. One type includes superior nodes, such as seismic monitoring nodes, which have superior data and alert data for transmission. The other type comprises ordinary nodes, such as picture-monitoring nodes, sound-monitoring nodes. Ordinary nodes have ordinary data to transmit. The operation of HT-MAC is divided into rounds, each of which consists of two stages: a set-up stage and a steady-state stage. To optimize energy consumption and maximize network lifetime, these two stages are performed alternately. At the beginning of one round, at the set-up stage, the nodes in this system are clustered according to their relative energy and location. Clustering can significantly improve the energy efficiency of the entire network. At the steady-state stage, the HT-MAC scheme is based on the distributed polling scheme. The timeline of HT-MAC at the steady-state stage is shown in Figure 2.

In this study, the protocol stack of ordinary and superior nodes includes physical, data link, network, transport, and application layers. Cooperation between layers is very important. The physical layer is responsible for the digital coding, modulation, filtering, and spreading of the signal. The physical layer of the protocol stack utilizes the direct-sequence spread–spectrum technique. The HT-MAC protocol is power-aware and can adjust the transmission power to save energy. The network layer protocol adopts static routing technology, and the nodes in the cluster send information to the cluster head, and then the cluster head converges and forwards the information to the base station. The transport layer is responsible for maintaining the flow of the superior, alert, and ordinary data. Different types of sensing tasks can be built and used in the application layer.

The operating procedures of the HT-MAC scheme in normal and abnormal situations are as follows.

### 4.1. Set-Up Stage

Clustering algorithms are very important for the network lifetime and delay performance of WSNs. The pLEACH algorithm is applied to cluster hierarchical sensor networks [46]. As previously mentioned, a collaborative disaster-monitoring system has a hierarchical architecture. The pLEACH algorithm is an adaptive clustering hierarchy algorithm with a high network lifetime and low communication delay. Based on centralized processing in the base station, the clustering algorithm first partitions the nodes into an optimal number of clusters and then selects the node with the highest energy in the cluster as the cluster head. In this study, we extend the pLEACH algorithm to heterogeneous sensor networks.

In heterogeneous sensor networks, the clustering method also pursues the maximum network lifetime; that is, the death of the first node is averaged in a round. Data aggregation and relays occur in the cluster head (CH); therefore, the cluster head nodes consume more energy than the other nodes. The rotation of the cluster head may balance the energy dissipation of the sensor nodes. The decision metric used to select the cluster heads is important. We define *T_rwtch_* as the residual working time of the CH, as a cluster head, for the basis of cluster head selection:

(1) $$T_{rwtch}=\frac{E_{r}}{P_{sc}+P_{ar}},$$

where *E_r_* is the residual energy of a sensor node, *P_sc_* is the mean power of sensing and calculation for this type of sensor node, and *P_ar_* is the mean power of data aggregation and relay as a cluster head for a sensor node. *T_rwtch_* indicates the persistent working time if a node is selected as a cluster head.

In this study, energy models similar to those proposed in [39] were used. The energy consumption of radio transmission can be calculated as follows:

(2) $$E_{Tx}(l,d)=\left\{\begin{array}{c} lE_{elec}+l\varepsilon_{fs}d^{2},\;d<d_{0}\\ lE_{elec}+l\varepsilon_{mp}d^{4},d\geq d_{0} \end{array}\right.,$$

where *E_Tx_*(*l*,*d*) is the energy of *l*-bit message transmission over distance *d*, *E_elec_* is the energy consumption for digital coding, modulation, filtering, and spreading of the signal, *ε_fs_* is the energy consumption of short distance transmission, and *ε_mp_* is the energy consumption of long distance transmission. In addition, *d*_0_ is the threshold of the distance and can be obtained when *d* = *d*_0_, in Equation (2):

(3) $$d_{0}=\sqrt{\frac{\varepsilon_{fs}}{\varepsilon_{mp}}}.$$

For receiving an *l* bit message, the radio expends

(4) $$E_{Rx}(l)=lE_{elec.}$$

The proposed clustering algorithm is a centralized cluster head selection scheme. Only the base station is responsible for the cluster division of the entire network. Base stations generally have a continuous power supply, high storage and computing power, and global network information. Therefore, a complex algorithm can be used to obtain optimized clustering results. The proposed clustering algorithm comprises two phases.

In the first phase, each sensor node sends its location and current energy to a base station using the CSMA/CA approach. According to this information, the base station obtains the optimal number of clusters [39]. Ordinary nodes are divided equally into sectors; thus, there are approximately equal numbers of ordinary nodes in each sector. Subsequently, all sensor nodes in a sector, including ordinary and superior nodes, form a cluster. The cluster head is selected from the ordinary nodes in a cluster. Therefore, the distance from any non-head node to the cluster head is less than the network radius, which reduces the overall communication energy consumption in the network.

In the second phase of the proposed clustering algorithm, the cluster head is selected. When the network is initialized, each sensor node sends a report message to the base station regarding its current energy and location. Based on these messages, the base station first calculates *T_rwtch_*, the residual working time, as a cluster head for each ordinary node. Subsequently, an ordinary node with the maximum *T_rwtch_* in a cluster is selected as the cluster head. Finally, the base station broadcasts information on the cluster heads and members of a cluster to all the sensor nodes. The information consists of the locations of the sensor nodes in a cluster; thus, the nodes can adjust their transmission power to fit the transmission distance within a cluster.

### 4.2. Steady-State Stage

In the steady-state stage, HT-MAC operates in the duty cycle mode. Each cluster has *M* superior nodes and *N* ordinary nodes, and one of the ordinary nodes is selected as the cluster head. The ordinary nodes in a cluster form a virtual ring, and the token runs in the ring. In one period, the ordinary node that owns the token first asks superior nodes if there are any data to transmit. If the superior nodes have data, they transmit the data sequentially from superior node 1 to superior node *M*. That is, in one period, the ordinary node with the token polls all the superior nodes individually in the cluster. Then, this ordinary node sends its own data to the destination node, which only transmits data frames that have already arrived when the node starts transmitting. Subsequently, this ordinary node passes the token to the next ordinary node, and the cluster enters the sleeping state. Considering the relatively short active state of the sensor nodes, the sleeping time accounts for a large proportion of the period time. During the sleeping state, the alert data of the superior nodes are transmitted using a low-power listening and shortened preamble approach to satisfy the delay and energy requirements of the alert. The low-power listening and shortened preamble approach was based on the X-MAC protocol [47]. The preamble sequence consists of many smaller shortened preambles with destination addresses. Using the time interval between the shortened preambles, the receiving node sends early acknowledgments to the source node. The sending node transmits the data packet immediately after receiving early confirmation to avoid excessive preambling of the sending node and overhearing of the receiving node, which reduces delays and energy consumption. A random back-off is performed to mitigate collisions between multiple sending nodes. Therefore, the alerts of superior nodes have a shorter communication delay, meeting the requirements of disaster-monitoring applications.

HT-MAC WSNs are composed of several clusters. To reduce the interference between adjacent clusters, each cluster in the HT-MAC scheme communicates based on a direct-sequence spread spectrum. Therefore, each cluster applies a unique spread code. In each cluster, the nodes deliver polling tokens individually, based on the virtual logical ring. In a virtual ring, a node’s predecessor is its previous node, and its successor is its next node. Consider a special situation in which there is only one node in the virtual ring: one node’s predecessor and successor.

For normal operation of the virtual ring, there are two basic procedures: a node may join or a node may leave a ring. First, a node joins a virtual ring. Each node in the virtual ring periodically transmits an invitation token that makes the node join the virtual ring. The procedure for joining a virtual ring is as follows. Node *m* in the ring first delivers an invitation token (*m*, *n*), where *n* denotes the successor node of node *m*. Node *m* invites a node to join between node *m* and *n,* using this invitation token. Upon receiving an invitation token, a node that intends to join the virtual ring checks whether it is within the coverage of the cluster. If confirmed, this node responds to the invitation of node *m,* and sets node *m* as the predecessor and node *n* as the successor. Node *m* sets this node as the successor, and node *n* sets this node as the predecessor. Therefore, this node joins the virtual ring. When multiple nodes want to join simultaneously, each node delays its response to the invitation token for a random time to avoid collisions. The node that sends the invitation token then determines which node will join the virtual ring, and delivers an acceptance token to that node for joining.

**Figure 3:**
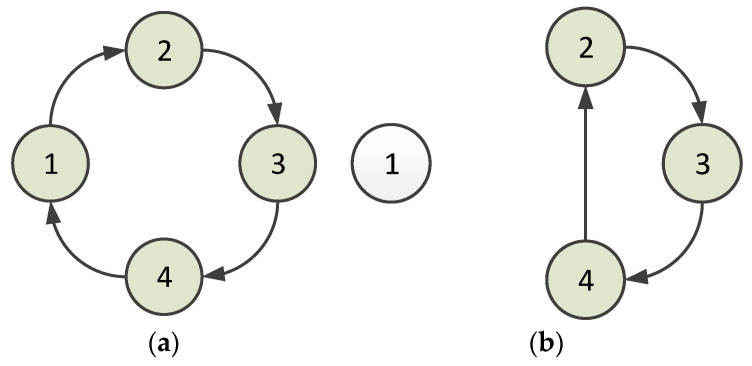
Situation when a node leaves a ring (1, 2, 3, and 4 denote nodes 1, 2, 3, and 4): (**a**) when Node 1 is about to leave the ring; (**b**) after Node 1 leaves.

Second, the node can leave the virtual ring. The polling token should be owned when a node is ready to leave the ring. If the polling token is passed to this node, it sends a message to its predecessor node to indicate that it is leaving. Consequently, the predecessor node allows this node to leave and gives the successor node a special token informing it that the predecessor has changed. This procedure is illustrated in Figure 3. Node 1 sends a message to Node 4 when Node 1 is about to leave the ring, as shown in Figure 3a; then, for setting up the predecessor to Node 4, a token for setting up the predecessor is passed from Node 4 to Node 2. Node 2 accepts this token and establishes Node 4 as its predecessor. Subsequently, Node 2 sends a token reply to Node 4, which establishes Node 2 as the successor. The situation after Node 1 leaves is shown in Figure 3b. In the case that multiple nodes leave the ring at the same time, since only the nodes that obtain the token can leave the ring network, the multiple nodes will leave the ring successively, according to the order in which the token is obtained.

The procedure for initiating a virtual ring is as follows. Each cluster has a cluster head node based on the proposed clustering method. Initially, the virtual ring has only one node, the cluster head node. The cluster head broadcasts an invitation token to join. One of the ordinary nodes in the cluster is verified as joining the virtual ring. This operation continues, and all ordinary nodes in the cluster finally join the virtual ring. The virtual ring initialization is complete.

### 4.3. Abnormity Handling

For reliable transmission in a disaster-monitoring system, the operating mode under abnormal situations should be considered in HT-MAC. In the following, we analyze abnormal situations and describe the handling methods based on the previous work [48].

**Figure 4:**
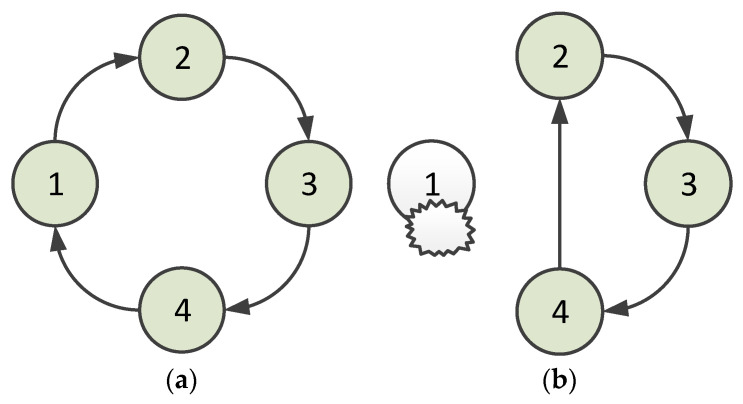
Situation in which a node is unavailable (1, 2, 3, and 4 denote nodes 1, 2, 3, and 4): (**a**) the normal ring; (**b**) after abnormity handling.

When a node is abnormal and unavailable, the situation is shown in Figure 4. Figure 4a shows the normal ring. When a node leaves the neighbor node’s coverage, for example, in the case in which Node 1 leaves the coverage of Node 4, Node 4 is responsible for handling the situation. In the HT-MAC scheme, a node receives an acknowledgment frame to confirm that its successor accepts the polling token. Hence, Node 1 begins transmitting data once its acknowledgment frame is received by Node 4. If the acknowledgment frame is not received, Node 4 continues to deliver a polling token to Node 1. After several attempts, Node 4 drops delivering the polling token. At this point, the virtual ring is opened. In such a situation, Node 4 delivers a token to arrange for Node 2 to be its new successor. When Node 2 accepts the token, it sets Node 4 as its predecessor and delivers a token reply to Node 4. Node 4 accepts the reply and sets Node 2 as its successor. This virtual ring is then closed, as shown in Figure 4b. The algorithm for handling abnormalities when a node is unavailable is presented in Algorithm 1.

In the case of a node moving out of coverage, the aforementioned operating procedure is also applicable. In fact, because the successor node cannot differentiate between failure, quitting, and interference from link noise, Algorithm 1 can be applied to all these cases.

**Figure 5:**
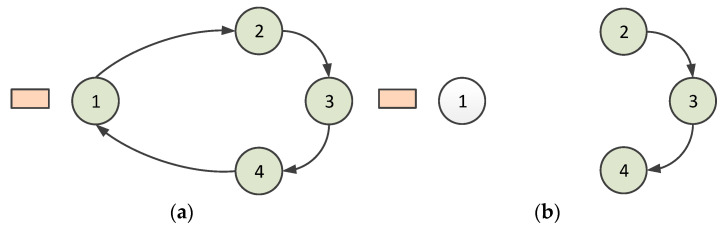
Situation without a token in the ring (1, 2, 3, and 4 denote nodes 1, 2, 3, and 4): (**a**) when Node 1 owns the polling token; (**b**) after Node 1 departs from the coverage of all the other nodes.

For the abnormal situation of a no-polling token in a virtual ring, efforts must be made to bring the virtual ring to work. This is illustrated in Figure 5a. At that moment, Node 1 departs from the coverage of all the other nodes; meanwhile, Node 1 owns the polling token, as shown in Figure 5b. In such a situation, there are no active polling tokens in the network. In the HT-MAC scheme, if a node has not received a polling token within the maximum period time, it generates a new token to deliver. Consequently, each node ultimately creates a new polling token. Therefore, multiple tokens exist in the ring simultaneously. This case is processed as follows.
**Algorithm 1.** Abnormity handling when a node is unavailable. **Input:** current node—current node with the polling token **Output:** current node—current node with the new successor node1A node delivers the polling token to its successor node;2**if** this node does not receive the token acknowledgement frame from the successor node3 This node continues to deliver the polling token to the successor node several times;4 **if** these retransmissions all fail5  This node delivers the token for setting up a new successor to the next node of the successor node;6  In the next node of the successor, the token is received and the predecessor node is set up with the current node;7  The new successor node is set up with the next node of the successor;8  The ring is reconfigured;9 **end if**10**end if**11**End**

**Figure 6:**
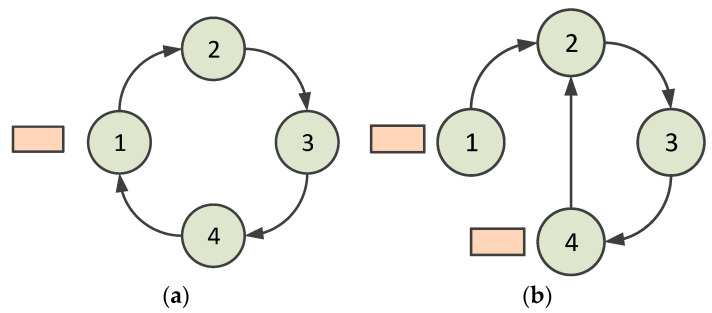
Situation with multiple tokens (1, 2, 3, and 4 denote nodes 1, 2, 3, and 4): (**a**) the normal situation; (**b**) with multiple tokens.

This method can remove multiple polling tokens, as shown in Figure 6. In HT-MAC, each polling token frame contains two parts: a token identifier and a sequence number. The token maker is a node that generates a polled token. The token identifier is determined by the MAC addresses of the token maker node. The token maker produces an integer as the polling token sequence number and increases it by one each time the token passes through the maker.

Based on the received polling token, the sequence number and token identifier are recorded by each node in the ring. Whenever a new polling token arrives, the node checks whether the sequence number of the new token is less than that of the previous token. If this is the case, the token is deleted by the node. Meanwhile, the node checks the received token for whether the sequence number of this token is the same as the previous one, and whether the token identifier is less than the previous token. If this is the case, the token will also be deleted by the node. As shown in Algorithm 2, this method guarantees that only one token exists in a virtual ring.
**Algorithm 2.** Abnormity handling of multiple tokens. **Input:** token maker—token with the ring address and token sequence number **Output:** current node—deleted token1Token maker produces the ring address;2Token maker sets the token sequence number by adding one every time when it passes the token maker;3The nodes in the ring record the token identifier and sequence number of the polling token;4A node receives a new polling token;5**if** the sequence number is less than the previous one6 Delete the received polling token;7**else**8 **if** the token identifier is less than the previous token9  Delete the polling token received;10 **else**11  This node transmits the data;12  In this node, the polling token is transmitted to the successor node;13 **end if**14**end if**15**End**

## 5. Theoretical Analysis

**Figure 7:**
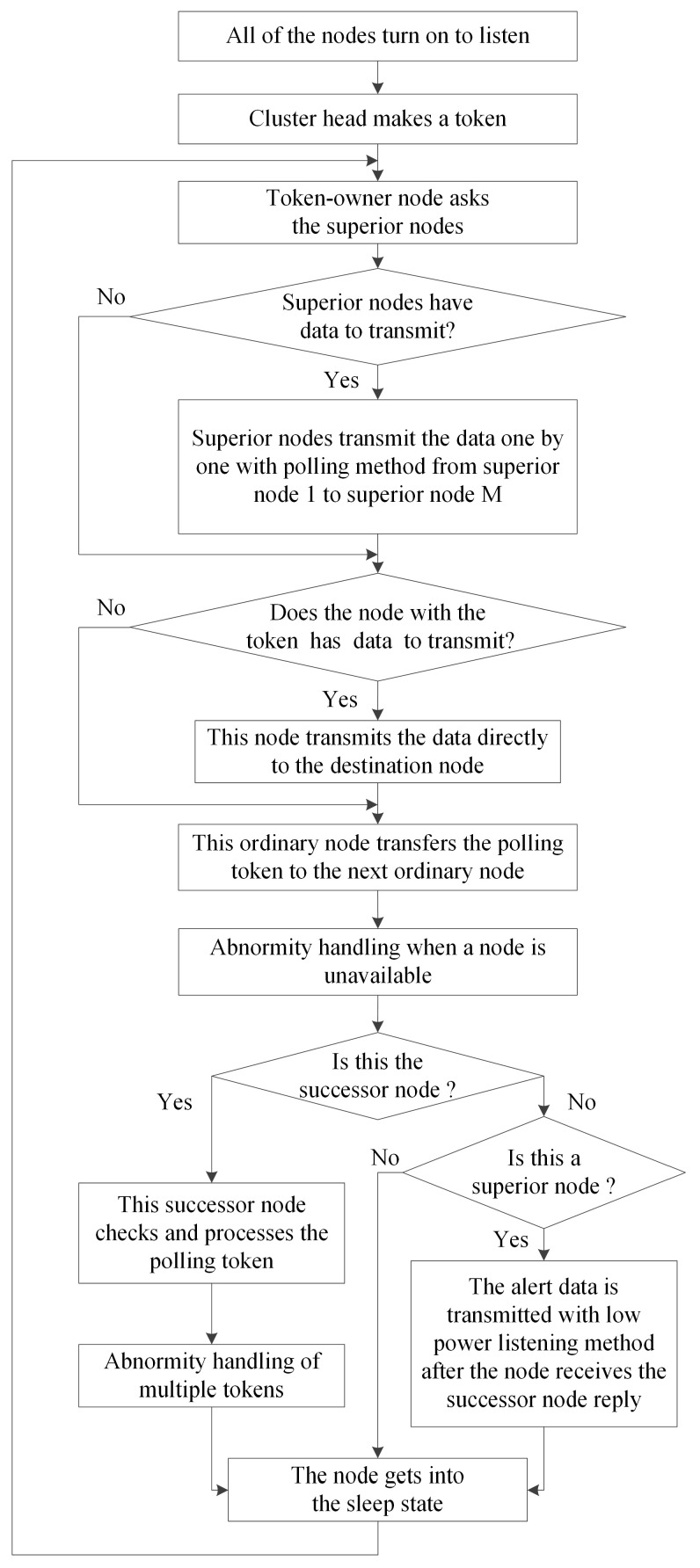
Flow chart of the HT-MAC scheme at the steady-state stage.

The details of the HT-MAC scheme in the steady-state stage are shown in Figure 7. HT-MAC is based on the duty cycle. After clustering, all nodes in one cluster begin to listen. A polling token is created and owned by the cluster head node. Subsequently, the token owner node asks the first superior node whether there are data to transmit. If the superior node has data to transmit, it simultaneously transmits the data directly to the destination node. The token owner node then asks the second superior node to transmit the data. This polling among the superior nodes is performed until the last superior node, superior node *M*. Subsequently, an ordinary node with a polling token determines whether there are data to transmit. If there are data, they are transmitted to the destination node directly. The node with the polling token then transfers the polling token to the next ordinary node in the virtual ring, and the algorithm for handling abnormalities when a node is unavailable is performed. The other nodes receive polling tokens and determine whether they are successor nodes. If there is a successor node, it obtains the polling token and checks if the token is corrupted. If the token is corrupted, the successor node will discard this token and deal with the abnormality using Algorithm 1; otherwise, the token reply will be sent. Then, the successor node performs abnormity handling of multiple tokens and enters the sleeping state. After a superior node receives a token reply from an ordinary node and there are alert data in this superior node, the superior node transmits the alert data at once under the low-power listening and shortened preamble approach. Otherwise, the superior node enters a sleeping state. During the sleeping state, if an alert appears in one superior node, the alert awakens the superior node and is transmitted at once, using the low-power listening and shortened preamble approach. The next period starts at the end of the sleeping state. Therefore, the polling token is transferred in a virtual token ring network. In the following sections, to facilitate theoretical analysis, we assume that there are two superior nodes within one cluster.

We assume that the ring network includes *N* + 2 nodes, and that there are two superior nodes and *N* ordinary nodes. Every node follows the first come, first served queue principle, and assumes that the queue capacity is unlimited. All the nodes provide a gated service; in other words, they send the arrived frames only when it is their turn to transmit. In ordinary node *i* (*i* = 1, 2, …, *N*), queue *i* (*i* = 1, 2, …, *N*) represents the queue of node *i*. Node *i* has a Poisson process input, with an arrival rate of *λ_i_*. The service time for the frames transmitted by an ordinary node is independent. The distribution function of the service time in an ordinary node *i* is *H_i_*(*x*). *P*1 and *P*2 represent the queues of superior Nodes 1 and 2, respectively. The input of the superior nodes is a Poisson process, and in superior Nodes 1 and 2, the arrival rates of the Poisson process are *λ_P_*_1_ and *λ_P_*_2_, respectively. The service time for the frames transmitted by a superior node is independent. The probability distribution of the frame transmission time is a general distribution in superior Nodes 1 and 2, and the distribution functions are *H_P_*_1_(*x*) and *H_P_*_2_(*x*), respectively. In the period from *t_n_* to *t_n_*_+1_, as shown in Figure 2, the data frame transmission time of ordinary node *i* is *τ_i_*, the sleep time is *s_i_*, and the polling token walking time is *μ_i_*. Within time *t*, the number of frames arriving in an ordinary node queue *i* is *υ_i_*(*t*). When the token ring system reaches a stable state, the instant at which ordinary node *i* begins to transmit frames, the probability of ordinary node *k* with *j_k_* frames waiting is *g_i_*(*j*_1_, *j*_2_, …, *j_P_*_1_, *j_P_*_2_). In ordinary nodes, assume that the instant when an ordinary node begins to transmit the data is …, *t_n_*, *t_n_*_+1_, …, so that … < *t_n_* < *t_n_*_+1_ < …. According to the random process, random variables may be defined as follows: *ε_n_*(*i*) is the frame number in ordinary node *I* when the very moment is *t_n_*; *ε_n_* is the ordinary node identifier when ordinary node *i* starts transmitting packets in the period from *t_n_* to *t_n_*_+1_; in ordinary node *i*, *h_i_* is the mean frame transmission time; and *h_P_*_1_ and *h_P_*_2_ are the mean frame transmission times of superior nodes *P*1 and *P*2, respectively. Subsequently, the state of the system at *t_n_* is described by (*ε_n_*, *ε_n_*(1), *ε_n_*(2), …, *ε_n_*(*N*), *ε_n_*(*P*1), *ε_n_*(*P*2)); meanwhile, the state space of the system becomes *I* = {(*i*, *k*_1_, *k*_2_, …, *k_j_*, …,*k_N_*, *k_P_*_1_, *k_P_*_2_): *i* = 1, 2, …, *N*; *k_j_* = 0, 1, 2, …; *j* = 1, 2, …, *N*, *P*1, *P*2}. Hence, the transition probabilities with respect to the state (*ε_n_*, *ε_n_*(1), *ε_n_*(2), …, *ε_n_*(*N*), *ε_n_*(*P*1), *ε_n_*(*P*2)) construct an irreducible, aperiodic Markov chain. The Markov chain is ergodic, and the system can be assumed to be in statistical equilibrium. In this case, the limiting probabilities of the states (*ε_n_*, *ε_n_*(1), *ε_n_*(2), …, *ε_n_*(*N*), *ε_n_*(*P*1), *ε_n_*(*P*2)) are obtained as follows:

(5) $$\underset{n\to \infty }{\lim}\mathrm{Pr}\left\{\varepsilon_{n}=i,\varepsilon_{n}(k)=j_{k};\;k=1,2,\ldots ,N,P1,P2\right\}=g_{i}(j_{1},j_{2},\ldots ,j_{N},j_{P1},j_{P2}).$$

Equation (5) indicates that the number of frames in a node remains steady after a long time. For the existence of an equilibrium state, the necessary and sufficient conditions are as follows:

(6) $$\sum_{i=1}^{N}\rho_{i}+\rho_{P1}+\rho_{P2}<1\rho_{i}=\lambda_{i}h_{i},\rho_{P1}=\lambda_{P1}h_{P1},\rho_{P2}=\lambda_{P2}h_{P2},$$

where *ρ_i_* is the traffic intensity for ordinary node *i,* and *ρ_P_*_1_ and *ρ_P_*_2_ are the traffic intensities for superior nodes *P*1 and *P*2, respectively. The above equation shows that the system exists in an equilibrium state when the total traffic intensity of the system is less than 1.

*F_P_*_1_(*x*) and *F_P2_*(*x*) are the frame transmission times for superior nodes *P*1 and *P*2 at any instant *t_i_* with *x* frames, respectively. Between *t_n_* and *t_n_*_+1_, in superior nodes *P*1 and *P*2, the frame transmission times are *F_P_*_1_[*ε_n_*(*P*1)] and *F_P_*_2_[*ε_n_*(*P*2)], respectively.

Consequently, the superior and ordinary nodes have the following number of frames at instant *t_n+_*_1_:

(7) $$\left\{\begin{array}{l} \varepsilon_{n+1}(P1)=\upsilon_{P1}(\tau_{i}+\mu_{i}+s_{i})+\upsilon_{P1}\{F_{P1}[\varepsilon_{n}(P1)]\}+\upsilon_{P1}\{F_{P2}[\varepsilon_{n}(P2)]\}\\ \begin{array}{l} \varepsilon_{n+1}(P2)=\upsilon_{P2}(\tau_{i}+\mu_{i}+s_{i})+\upsilon_{P2}\{F_{P2}[\varepsilon_{n}(P2)]\}\\ \varepsilon_{n+1}(G_{i})=\upsilon_{G_{i}}(\tau_{i}+\mu_{i}+s_{i})\\ \varepsilon_{n+1}(G_{j})=\varepsilon_{n}(G_{j})+\upsilon_{G_{j}}(\tau_{i}+\mu_{i}+s_{i})+\upsilon_{G_{j}}\{F_{P1}[\varepsilon_{n}(P1)]\}+\upsilon_{G_{j}}\{F_{P2}[\varepsilon_{n}(P2)]\} \end{array} \end{array}\right..$$

From the assumption of Poisson arrivals of node data, the following relation holds if *τ_i_*, *μ_i_* and *s_i_* do not overlap:

(8) $$\upsilon (\tau_{i}+\mu_{i}+s_{i})=\upsilon (\tau_{i})+\upsilon (\mu_{i})+\upsilon (s_{i}).$$

Therefore, the generating function of the transition probability Pr(*i* + 1, *ε_n_*_+1_(1), *ε_n_*_+1_(2), …, *ε_n_*_+1_(*N*), *ε_n_*_+1_(*P*1), *ε_n_*_+1_(*P*2)) is as follows:

(9) $$E[\prod_{k=1}^{N}x_{G_{k}}^{\varepsilon_{n+1}(G_{k})}\cdot x_{P1}^{\varepsilon_{n+1}(P1)}\cdot x_{P2}^{\varepsilon_{n+1}(P2)}]=U_{i}^{*}(A)\cdot S_{i}^{*}(A)\cdot E[\prod_{k\neq i}x_{G_{k}}^{\varepsilon_{n}(G_{k})}\cdot {\left[H_{i}^{*}(A)\right]}^{\varepsilon_{n}(i)}\cdot {\left[H_{P1}^{*}(B)\right]}^{\varepsilon_{n}(P1)}\cdot {\left[H_{P2}^{*}(C)\right]}^{\varepsilon_{n}(P2)}],$$

where *A*, *B*, and *C* are given by the following:

(10) $$\left\{\begin{array}{l} A=\sum_{k=1}^{N}\lambda_{G_{k}}(1-x_{G_{k}})+\lambda_{P1}(1-x_{P1})+\lambda_{P2}(1-x_{P2})\\ B=\sum_{k\neq i}\lambda_{G_{k}}(1-x_{G_{k}})+\lambda_{P1}(1-x_{P1})\\ C=\sum_{k\neq i}\lambda_{G_{k}}(1-x_{G_{k}})+\lambda_{P1}(1-x_{P1})+\lambda_{P2}(1-x_{P2}) \end{array}\right..$$

Letting *n*→∞ on both sides of Equation (9), we obtain the generating function of the transition probability Pr(*i* + 1, *ε_n_*_+1_(1), *ε_n_*_+1_(2), …, *ε_n_*_+1_(*N*), *ε_n_*_+1_(*P*1), *ε_n_*_+1_(*P*2)) is *G_i_*(*x*_1_, *x*_2_ …, *x_N_*, *x_P_*_1_, *x_P_*_2_) as follows:

(11) $$G_{i+1}(x_{1},x_{2},\ldots ,x_{N},x_{P1},x_{P2})=U_{i}^{*}(A)\cdot S_{i}^{*}(A)\cdot G_{i}(x_{1},x_{2},\ldots ,H_{i}^{*}(A),\ldots ,x_{N},H_{P1}^{*}(B),H_{P2}^{*}(C)).$$

In the above equation, when the polling token moves forward during the period from *t_n_* to *t_n_*_+1_, $U_{i}^{*}(s)$(*i* = 1,2, …, *N*) is the Laplace–Stieltjes transform of the walking time probability distribution; $S_{i}^{*}(s)$ is the Laplace–Stieltjes transform of the sleeping time probability distribution when the polling token moves forward from *t_n_* to *t_n_*_+1_. In ordinary node *i* with a Poisson input, *H_i_*(*x*) represents the busy period probability distribution, and $H_{i}^{*}(s)$ is the Laplace–Stieltjes transform of *H_i_*(*x*). In superior nodes *P*1 and *P*2 with a Poisson input, *H_P_*_1_(*x*) and *H_P_*_2_(*x*) represent the busy period probability distributions for *P*1 and *P*2, respectively, and $H_{P1}^{*}(s)$ and $H_{P2}^{*}(s)$ are the Laplace–Stieltjes transforms of *H_P_*_1_(*x*) and *H_P_*_2_(*x*), respectively. Using the above recursive formula, *G_i_*(*x*_1_, *x*_2_ …, *x_N_*, *x_P_*_1_, *x_P_*_2_) can be described as a functional equation; however, an explicit representation cannot be derived.

We denote *g_i_*(*j*) as the mean node queue length in queue *j* (*j* = 1, 2, …, *N*, *P*1, *P*2), when ordinary node *i* begins to transmit data, where *i =* 1, 2, …, *N*:

(12) $$g_{i}(j)=\underset{x_{1},x_{2},\ldots ,x_{N},x_{P1},x_{P2}\to 1}{\lim}\frac{\left\{\partial G_{i}(x_{1},x_{2},\ldots ,x_{N},x_{P1},x_{P2})/\partial x_{j}\right\}}{G_{i}(1,1,\ldots ,1,1,1)}.$$

From Equation (11) for *i =* 1, 2, …, *N*, we set:

(13) $$G_{i}(1,1,\ldots ,1,1,1)=K\;(\mathrm{constant})\;i=1,2,\ldots ,N.$$

In Equation (11), the differential operation of *x_j_* and *x_l_*→1 (*l* = 1, 2, …, *N*, *P*1, *P*2) yields

(14) $$\left\{\begin{array}{l} g_{i+1}(j)=\lambda_{j}\mu_{i}+\lambda_{j}s_{i}+g_{i}(j)+\lambda_{j}h_{i}g_{i}(i)+\lambda_{j}h_{P1}g_{i}(P1)+\lambda_{j}h_{P2}g_{i}(P2)\\ g_{i+1}(i)=\lambda_{i}\mu_{i}+\lambda_{i}s_{i}+\lambda_{i}h_{i}g_{i}(i)\\ g_{i+1}(P1)=\lambda_{P1}\mu_{i}+\lambda_{P1}s_{i}+\lambda_{P1}h_{i}g_{i}(i)+\lambda_{P1}h_{P1}g_{i}(P1)+\lambda_{P1}h_{P2}g_{i}(P2)\\ g_{i+1}(P2)=\lambda_{P2}\mu_{i}+\lambda_{P2}s_{i}+\lambda_{P2}h_{i}g_{i}(i)+\lambda_{P2}h_{P2}g_{i}(P2) \end{array}\right..$$

The above equation represents the relationship between the mean queue lengths of the different nodes. We then combine the first and second equations in Equation (14) with *i* = *j*, *j* + 1, …, *i* − 1, *i*, and the following is obtained:

(15) $$g_{i+1}(j)=\lambda_{j}\mu_{j}+\lambda_{j}s_{j}+\lambda_{j}h_{j}g_{j}(j)+\sum_{k=j+1}^{i}\left[\lambda_{j}\mu_{k}+\lambda_{j}s_{k}+\lambda_{j}h_{k}g_{k}(k)+\lambda_{j}h_{P1}g_{k}(P1\right)+\lambda_{j}h_{P2}g_{k}(P2)],$$

where, when *j* > *i*, the following is attained:

(16) $$\sum_{k=j}^{i}=\sum_{k=j}^{N}+\sum_{k=1}^{i}.$$

When the ordinary nodes in the token ring network are symmetric, that is, *λ_i_* = *λ*, *μ_i_* = *μ*, *h_i_* = *h*, and *s_i_* = *s*, and the system is in the equilibrium state, the following can be set:

(17) $$\left\{\begin{array}{l} g(P1)=g_{i+1}(P1)=g_{i}(P1)\\ g(P2)=g_{i+1}(P2)=g_{i}(P2)\\ g=g_{j}(j) \end{array}\right..$$

With Equations (14)–(17), the following is obtained:

(18) $$g(P2)=\frac{\lambda_{P2}(\mu +s+hg)}{\left(1-\lambda_{P2}h_{P2}\right)}.$$

Additionally, *g*(*P*1) is given by the following:

(19) $$g(P1)=\frac{\lambda_{P1}(\mu +s+hg)}{\left(1-\lambda_{P1}h_{P1})(1-\lambda_{P2}h_{P2}\right)}.$$

From Equations (14), (18) and (19), we obtain

(20) $$g=\frac{\left(\lambda \mu +\lambda s)(N-\rho_{P1}-\rho_{P2}+\rho_{P1}\rho_{P2}\right)}{\left(1-\rho_{P1})(1-\rho_{P2})-\rho (N-\rho_{P1}-\rho_{P2}+\rho_{P1}\rho_{P2}\right)}.$$

Equation (20) is the mean queue length of node *k* when ordinary node *k* (*k* = 1, 2, …, *N*) begins to transmit data. Substituting Equation (20) into Equation (19) yields

(21) $$g(P1)=\frac{\lambda_{P1}(\mu +s)}{\left(1-\rho_{P1})(1-\rho_{P2})-\rho (N-\rho_{P1}-\rho_{P2}+\rho_{P1}\rho_{P2}\right)}.$$

From Equations (18) and (20), *g*(*P*_2_) can be expressed as

(22) $$g(P2)=\frac{\lambda_{P2}(1-\rho_{P1})(\mu +s)}{\left(1-\rho_{P1})(1-\rho_{P2})-\rho (N-\rho_{P1}-\rho_{P2}+\rho_{P1}\rho_{P2}\right)}.$$

When an ordinary node *k* (*k* = 1, 2, …, *N*) begins transmitting data, Equations (21) and (22) are the mean queue lengths of superior nodes *P*_1_ and *P*_2_, respectively. Thereafter, we can derive the mean cycle time of the token ring network when the ordinary nodes are symmetric and the system is in an equilibrium state:

(23) $$T_{c}=N\cdot [\mu +s+hg+h_{P1}g(P1)+h_{P2}g(P2)].$$

Substituting Equations (20)–(22) into the above equation, the following is obtained:

(24) $$T_{c}=\frac{N(\mu +s)}{\left(1-\rho_{P1})(1-\rho_{P2})-\rho (N-\rho_{P1}-\rho_{P2}+\rho_{P1}\rho_{P2}\right)}.$$

Equation (24) represents the mean cycle time of the HT-MAC scheme, and the mean cycle time indicates the extreme case of frame delay in a virtual ring network. Because the nodes are comparatively close to each other, the radio propagation delay can be ignored. In the worst case, a frame waits for one period to be transmitted to an ordinary node; therefore, the mean upper bound of the frame delay in the ordinary node is as follows:

(25) $$T_{ub\_on}=\frac{N(\mu +s)}{\left(1-\rho_{P1})(1-\rho_{P2})-\rho (N-\rho_{P1}-\rho_{P2}+\rho_{P1}\rho_{P2}\right)}+h.$$

We can judge the frame delay in the system using the mean upper bound to satisfy the delay requirement. Unlike an ordinary node, a superior node has a higher priority; therefore, it has the chance to transmit frames in each period. Thus, the mean upper bound of the frame delay in superior Node 1 can be derived as follows:

(26) $$T_{ub\_P1}=\frac{\left(\mu +s\right)}{\left(1-\rho_{P1})(1-\rho_{P2})-\rho (N-\rho_{P1}-\rho_{P2}+\rho_{P1}\rho_{P2}\right)}+h_{P1}.$$

Additionally, the mean upper bound of the frame delay in superior Node 2 can be derived as follows:

(27) $$T_{ub\_P2}=\frac{\left(\mu +s\right)}{\left(1-\rho_{P1})(1-\rho_{P2})-\rho (N-\rho_{P1}-\rho_{P2}+\rho_{P1}\rho_{P2}\right)}+h_{P2}.$$

The mean queue length, mean cycle time, and mean upper bound of frame delay for the HT-MAC scheme are obtained. Next, we analyzed the performance of the proposed scheme. The theoretical results and investigations of the HT-MAC scheme were verified through simulations.

## 6. Performance Evaluation

In this section, we evaluate the performance of the HT-MAC scheme and compare it with those of other protocols. With Equations (20)–(22) and (24)–(27), the theoretical mean queue length, mean cycle time, and mean upper bound of the frame delay are obtained for the HT-MAC scheme. A computer simulation model of HT-MAC based on Network Simulator-2 (NS-2) was established to verify the theoretical results and evaluate scheme performance. NS-2 is an object-oriented discrete event simulator that covers a large number of network types, elements, protocols, applications, traffic models, and energy models. HT-MAC performance was evaluated in two parts: clustering performance and data transmission performance.

In the simulations, we deployed *N* = 100 ordinary nodes and *M* = 10 superior nodes that were randomly distributed in a 500 m × 500 m field. Ordinary nodes consisted of picture- and sound-monitoring nodes, and these two types of nodes each accounted for 50% of the total number of ordinary nodes. Without loss of generality, we assume that the base station is located at the center of the sensing field. In addition, the link rate was 11 Mb/s, the token frame length was 8 bytes, and for the superior and ordinary nodes, the mean data frame length was 256 bytes.

### 6.1. Clustering Performance

The proposed clustering method was compared with the pLEACH algorithm using the same radio model and simulation parameters. The message sizes sent by the sound-monitoring, seismic monitoring, and picture-monitoring nodes to the cluster head in each round were 5, 15, and 25 kB. The initial energies of the sound-, seismic, and picture-monitoring nodes were 2 J, 3 J, and 5 J, respectively. The other simulation parameters of the proposed model are listed in Table 3.

In HT-MAC, *T_rwtch_*, the residual working time as a cluster head, is the variable that determines which node is the cluster head. We assume that a sensor node gains *l* bits of sensing data in time *t_sc_*; thus, *P_sc_* the mean power of sensing and calculation for this node is

(28) $$P_{sc}=\frac{lE_{sc}}{t_{sc}},$$

where *E_sc_* is the energy consumption for sensing and calculating this type of node. If a sensor node aggregates *k* bits of data and relays them to a base station at a distance *d* in time *t_ar_*, *P_ar_* is the mean power of the data aggregation and relay as a cluster head:

(29) $$P_{ar}=\frac{kE_{DA}+kE_{Tx}(k,d)}{t_{ar}}.$$

**Figure 8:**
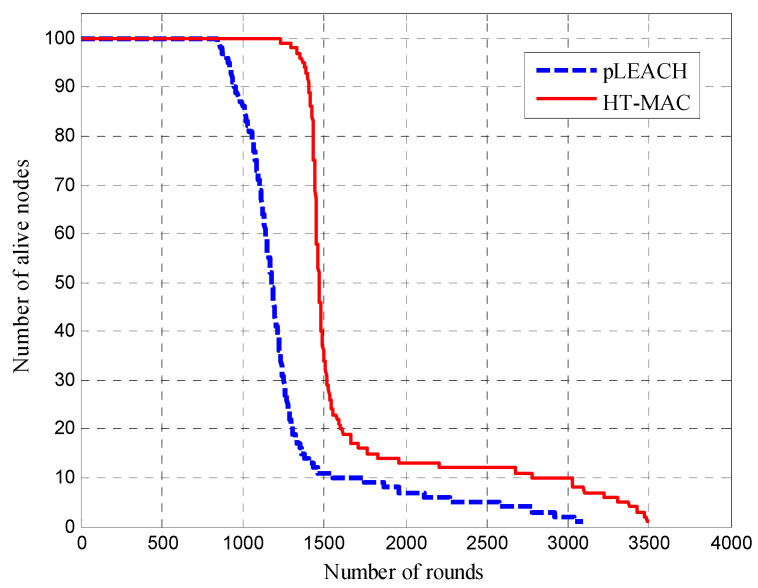
Number of nodes alive over time.

Through clustering simulations, the number of alive nodes for pLEACH and HT-MAC are is in Figure 8. At the same number of rounds, HT-MAC had more live nodes than pLEACH. Owing to the uneven energy consumption, pLEACH started to experience node energy exhaustion at over 847 rounds. However, HT-MAC did not experience node energy exhaustion until more than 1227 rounds. After experiencing a rapid decline, the number of live HT-MAC nodes gradually stabilized at approximately 1700 rounds. When the number of members in a cluster decreases, the energy consumption of the cluster head for relaying decreases significantly. A similar phenomenon was observed with pLEACH.

**Figure 9:**
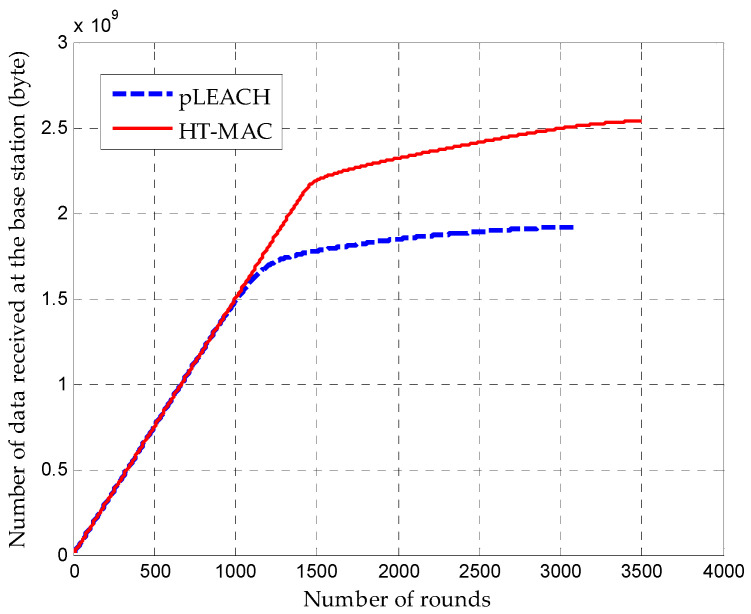
Total amount of data received at the base station over time.

The total amount of data received at the base station during the network lifetime is shown in Figure 9. This shows that the amount of received data increases smoothly at the beginning for HT-MAC and pLEACH. After 847 rounds, HT-MAC received significantly more data at the base station than the pLEACH algorithm, and the growth of the data received by pLEACH became slower. The data received by HT-MAC also become slower after 1227 rounds, but HT-MAC still receives much more data than the pLEACH algorithm, and it perseveres longer.

### 6.2. Data Transmission Performance

**Figure 10:**
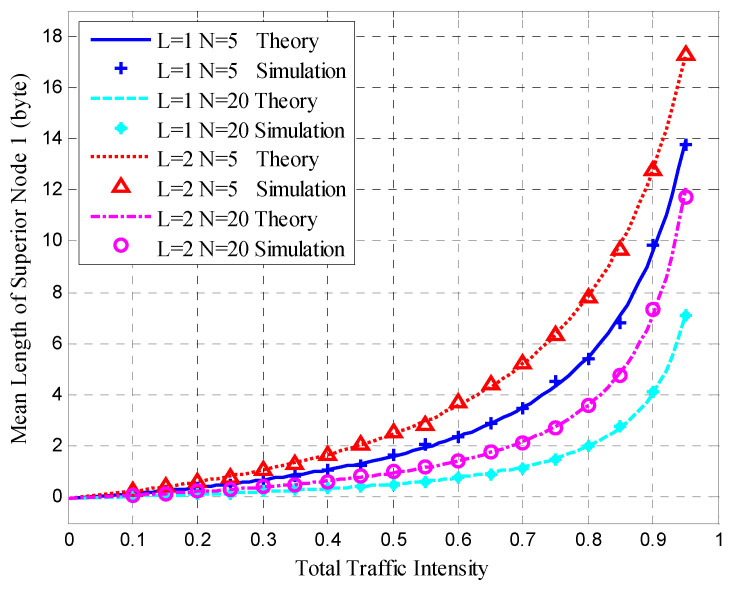
Mean queue length of superior Node 1.

**Figure 11:**
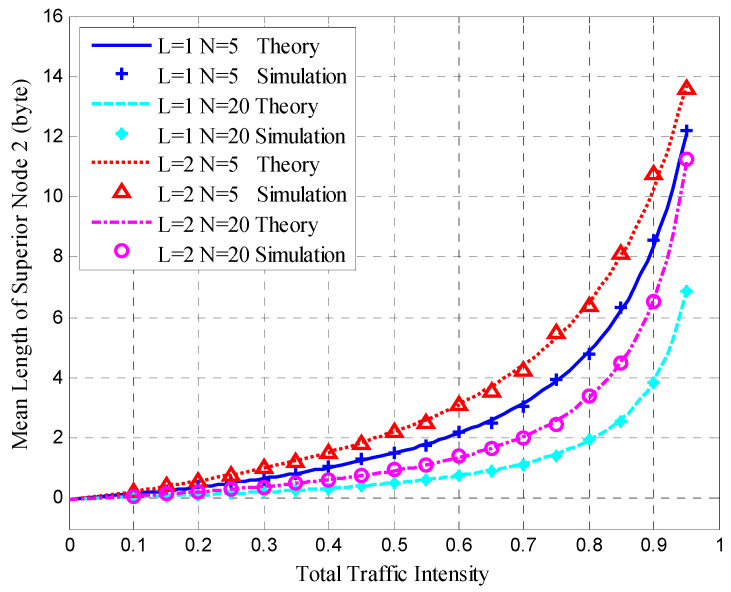
Mean queue length of superior Node 2.

**Figure 12:**
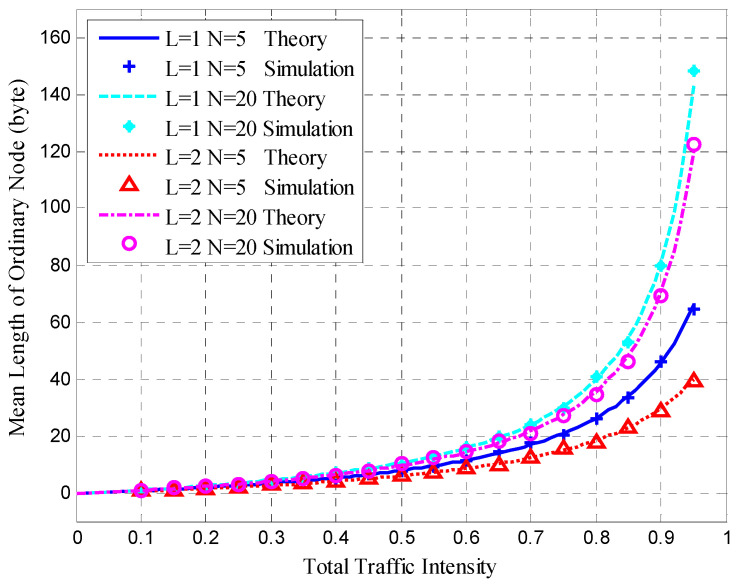
Mean queue length of the ordinary node.

**Figure 13:**
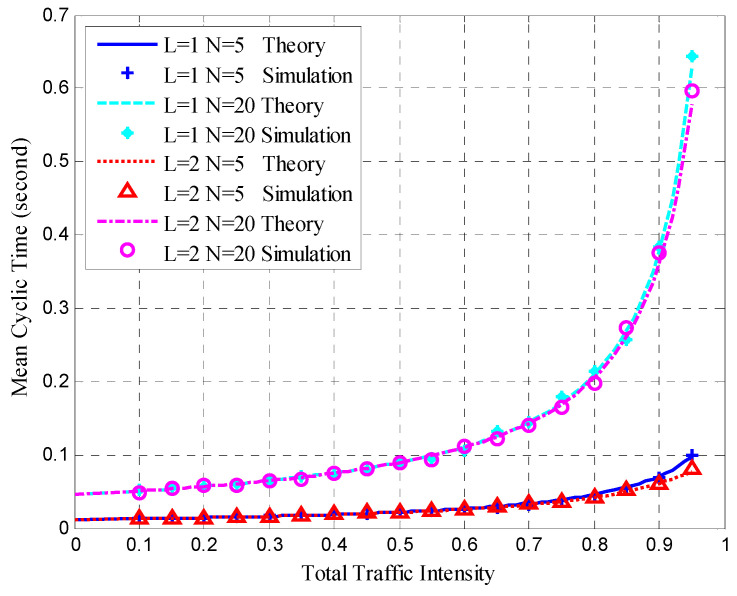
Mean cycle time.

**Figure 14:**
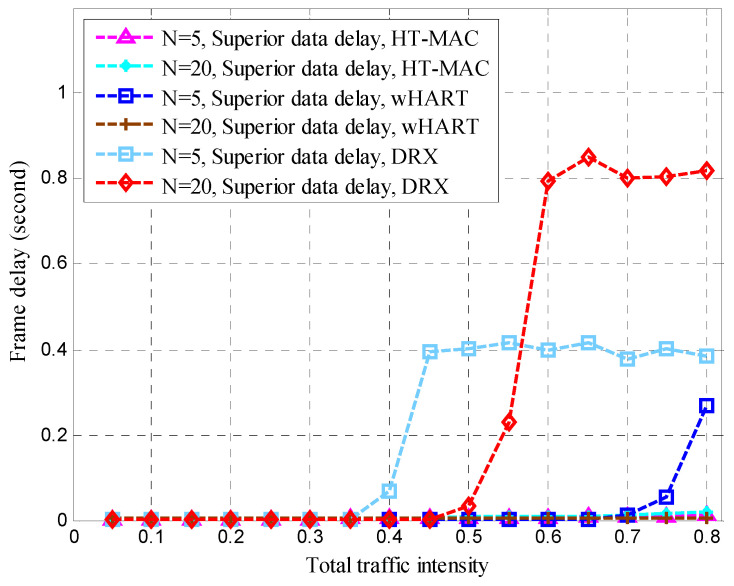
Superior data delay.

**Figure 15:**
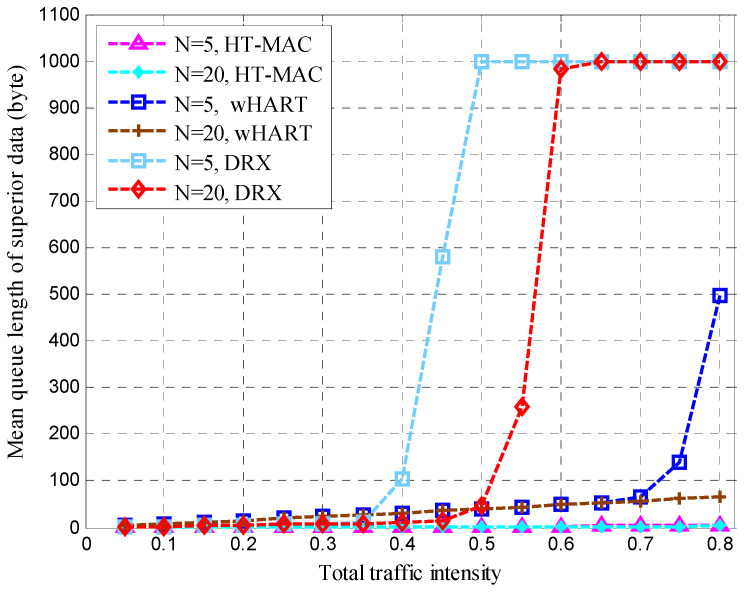
Mean queue length of superior data in superior node.

**Figure 16:**
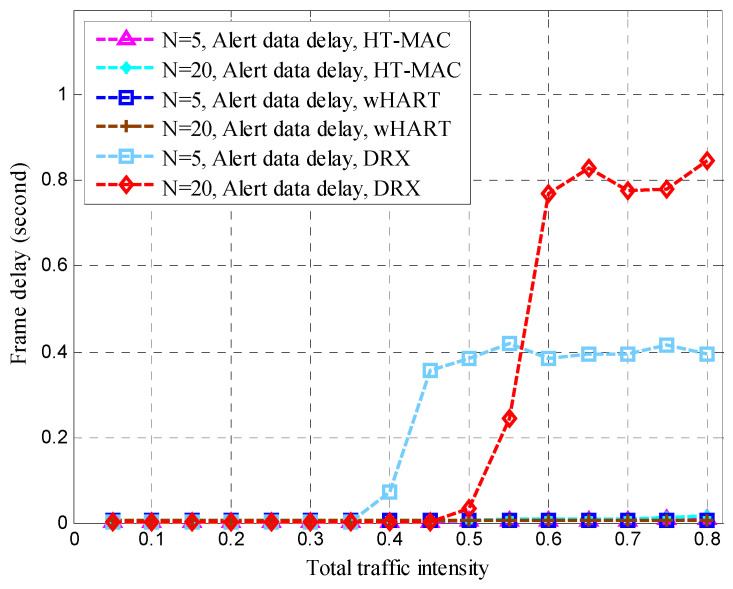
Alert data delay.

**Figure 17:**
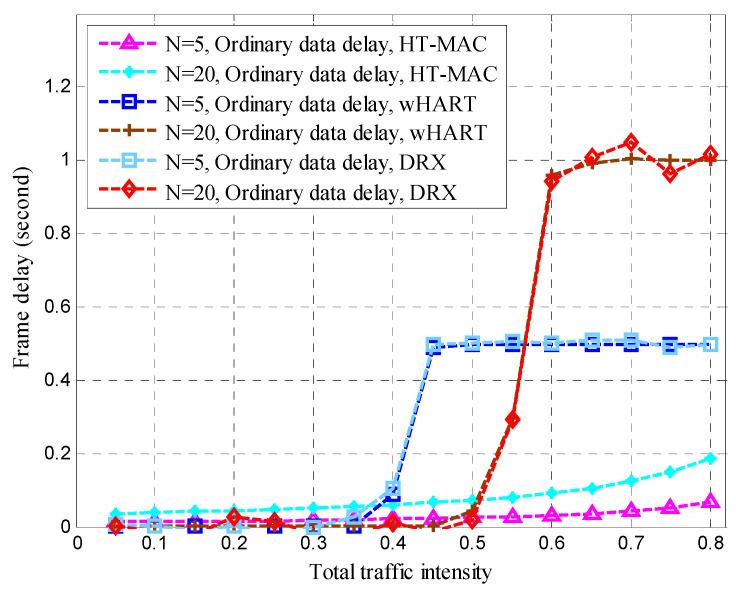
Ordinary data delay.

After clustering, the nodes transmitted sensing data at the steady-state stage in each cluster. The mean queue length, mean cycle time, and mean upper bound of the frame delay were obtained for the HT-MAC scheme. We verified the results of the theoretical analysis and studied the system performance as shown in Figure 10, Figure 11, Figure 12, Figure 13, Figure 14, Figure 15, Figure 16 and Figure 17.

The simulations used the same parameters as in the theoretical analysis, and the system was assumed to have 5, 10, and 20 ordinary nodes in a cluster. Each cluster consists of two superior nodes. We compared the simulation and theoretical results with the change in total traffic intensity under different conditions. The total traffic intensity is the ratio of all node loads (both ordinary and superior nodes) to the link rate. The total traffic intensity range is from 0.1 to 0.95 in theoretical calculations and simulations, with a step of 0.05. Ordinary nodes have symmetrical inputs, the traffic intensity proportion of the superior node to the ordinary node is denoted by *L*, and the values are 1 and 2.

Figure 10 presents the mean queue length of superior Node 1, and shows that the simulation results agree with the theoretical results under different degrees of total traffic intensity. As shown in Figure 10, when the total traffic intensity increases, the mean queue length of superior node 1 also increases. When the load proportion *L* is the same, more nodes in the cluster lead to a smaller mean queue length for the superior node. In fact, more nodes in the cluster cause a lower traffic intensity for superior node 1 under the same total traffic intensity. When the number of nodes is the same (*N* = 5) and *L* is larger, the mean length of the superior node increases.

Figure 11 presents the mean queue length of superior Node 2 and shows that the simulation results conform to the theoretical results under different degrees of total traffic intensity. Figure 11 is similar to Figure 10; however, the mean queue length of superior Node 2 is less than that of superior Node 1 under the same configuration.

Figure 12 shows the mean queue length of ordinary nodes, which agrees with the theoretical results under different degrees of total traffic intensity. This verified the accuracy of the theoretical analysis. In Figure 12, when the total traffic intensity exceeds 0.8, the mean queue length of ordinary nodes increases substantially.

Figure 13 shows the mean cycle times for the different situations. The simulation and theoretical results were in agreement under various conditions. Figure 13 shows that the mean cycle time is similar when the number of nodes and total traffic intensity are the same. With *N* = 5, the mean cycle time increases when the total traffic intensity exceeds 0.8. When *N* = 20 and the total traffic intensity exceeds 0.7, the mean cycle time increases rapidly.

Frame delays are vital in disaster-monitoring applications. As part of the WirelessHART simulation scenario, alert data were added to WirelessHART. Contention-free period slots were used for alert data, whereas contention access period slots were used for ordinary data. In the WirelessHART simulation, superior data were based on a contention-free period. We created a DRX simulation scenario and set the alert and superior data delay thresholds to 0.4 s. The traffic intensity proportion of the superior node to that of the ordinary node is 2. The queue buffers of the superior, ordinary, and alert data were 1 kB each. As shown in Figure 14, Figure 15, Figure 16 and Figure 17, the performance of HT-MAC is presented and compared with those of the WirelessHART and DRX protocols.

In the HT-MAC scheme, superior data frames are much faster than ordinary data frames. With increasing traffic intensity, superior data frame delays change only slightly, far less than the frame delay of ordinary data.

The mean queue length of the superior data at the superior node is shown in Figure 15. When *N* = 5, the mean queue length of the superior data for DRX starts to rise rapidly at a traffic intensity of 0.5, and reaches the maximum of the queue buffer at a traffic intensity of 0.65. At *N* = 20, the DRX protocol is similar to that at *N* = 5. When *N* = 5, the mean queue length of the superior data for WirelessHART starts to increase rapidly at a traffic intensity of 0.7. However, when *N* = 20, the mean queue length of the superior data for WirelessHART continues to increase slowly. When *N* = 5 and 20, HT-MAC always has a very short queue length for superior data.

Compared with the DRX scheme, HT-MAC has a smaller data-frame delay when the traffic intensity increases. As the traffic intensity increases above 0.4 (*N* = 5) and 0.5 (*N* = 20), DRX’s superior data frame delay increases rapidly. The results for WirelessHART were similar to those for DRX. When the traffic intensity exceeds 0.4 (*N* = 5), the superior data frame delay of WirelessHART increases rapidly.

HT-MAC’s alert data frame delay is clearly better than that of DRX, particularly when the total traffic intensity exceeds 0.4, and the alert data frame delay remains low and is smaller than that of WirelessHART. For *N* = 5, the maximum alert data frame delay of HT-MAC is 9 ms and 15 ms for *N* = 20. The ordinary data frame delay of HT-MAC also remains low when the total traffic intensity increases. However, when the total traffic intensity exceeds 0.4, the ordinary data frame delay of WirelessHART increases. When the total traffic intensity exceeds 0.5, the ordinary data frame delay of DRX increases. The mean upper bound of frame delay is an important performance metric. The simulation results showed that 99.23% of the frame delays satisfied the mean upper bound of the frame delay in an ordinary node, 99.57% satisfied the mean upper bound for superior Node 1, and 99.42% satisfied the mean upper bound for superior Node 2. For HT-MAC, the frame delay performance for the three types of data was better than those of the WirelessHART and DRX schemes.

Throughput is also a key indicator in disaster-monitoring applications. For *N* = 20, the mean throughput of the ordinary node is 364 kb/s, and that of the superior node is 732 kb/s. When *N* = 5, the mean throughput of the ordinary node is 973 kb/s, and that of the superior node is 1.9 Mb/s. In the HT-MAC scheme, the delay and throughput specifications for superior, alert, and ordinary data follow the requirements for crucial services such as earthquake surveillance.

## 7. Conclusions and Future Work

In disaster-monitoring scenarios, real-time accurate information with uninterrupted monitoring is crucial. In this study, we first present a collaborative disaster-monitoring system based on WSNs, and show the entire architecture of the system. Then, we obtain seismic data in a highly energy-efficient manner, based on the compressed sensing method.

In this study, a superior node hybrid scheme, HT-MAC, for disaster monitoring in wireless sensor networks is proposed, and the operating procedures of clustering and data transmission are presented. The proposed clustering algorithm for heterogeneous networks can effectively extend the network’s lifetime. HT-MAC operates in duty cycle mode at the steady-state stage, and ordinary nodes transmit multimedia data based on a virtual token ring. During each period, seismic data are promptly transmitted when each superior node is polled. Owing to the relatively short active state of the sensor nodes, the alert data are transmitted with a low-power listening and shortened preamble approach during the sleep state to satisfy the delay requirement. HT-MAC can simultaneously satisfy the requirements of three types of data for disaster-monitoring applications. These three types of data are ordinary data with high throughput, seismic data with high throughput and low delay, and alert data with high reliability and low delay of tens of milliseconds. HT-MAC must support reliable transmission, even in abnormal situations, and the handling procedures for abnormal situations are described in detail. Based on embedded Markov chains, a model of the HT-MAC scheme was developed, and the mean queue length, mean cycle time, and mean upper bound of the frame delay were obtained.

A simulation model of HT-MAC was built, and the simulation results verified the theoretical analysis. Using the simulation models of HT-MAC and pLEACH, the HT-MAC clustering algorithm significantly outperformed the pLEACH algorithm and increased the lifetime of the network. Based on the simulations of WirelessHART and DRX, the frame delay performance of HT-MAC was better than that of WirelessHART and DRX. We found that the mean delay of the superior and alert data was far less than that of the ordinary data, and a low delay level was maintained, especially when the traffic intensity was heavy. The alert data have a maximum frame delay of 15 ms, and HT-MAC provided a data rate of several hundred kb/s for superior and ordinary data. These results satisfy the requirements of disaster-monitoring applications.

As the next step, we will establish a testbed to analyze and validate the HT-MAC scheme. Technological advances have enabled the development of disaster-monitoring systems [13]; collaborative disaster-monitoring systems can be widely used in earthquake early warning and rescue, volcanic eruption early warning and monitoring, landslide early warning, and other disaster-monitoring applications. In the future, we will implement collaborative disaster-monitoring systems in order to perform seismic monitoring. Practical application of HT-MAC may significantly improve our ability to cope with disasters.

## Figures and Tables

**Table 1:** MAC protocols for wireless sensor networks.

Classification	Protocol
Contention-based MAC	S-MAC, B-MAC, PMAC, RT-MAC, MaxMAC, ENCO, QoS-MAC, DRX, PW-MAC, CSMA/WSD
Schedule-based MAC	BMA, TRAMA, TDMA-W, ArDez, DMAC
Hybrid MAC	CSMA-STDMA, E-hybrid, WirelessHART, Z-MAC, TCS
Multiple wireless interfaces MAC	MMAC-HR, DSP

**Table 2:** Comparison of relevant MAC protocols.

Protocol	Main Scheme	Computation Overhead	Control Overhead	Adaptivity to Topology Changes	Delay	Time Synchronization Precision
S-MAC	Virtual cluster, adaptive listening	No	Rather high	Good	Rather high	Low
B-MAC	LPL, clear channel assessment	No	High	Good	Moderate	Moderate
PMAC	Pattern exchange, avoid overhearing	Pattern generation	High	Good	Moderate	Moderate
RT-MAC	Feedback control, avoid contending	Clear channel control	Rather high	Low	Low	High
MaxMAC	Extra wake-ups, traffic adaptation	Duty cycle control	High	Good	Moderate	Low
ENCO	Estimated number of contenders	Contention window sizes	Moderate	Good	Moderate	Low
QoS-MAC	Differentiated service	No	Rather low	Good	Moderate	Low
DRX	Priority- and delay-aware	No	Rather low	Good	Low	Low
PW-MAC	Predictive wakeup duty cycle	On-demand Prediction	Low	Good	Low	Moderate
CSMA/WSD	Weak signal detection	Packet loss diagnosis	Low	Poor	Moderate	Low
BMA	Clustered network, slot assignment	Slot assignment	High	Moderate	High	High
TRAMA	Winning slot, reservation, piggy back	Transmission priority	High	Moderate	High	High
TDMA-W	Graph-coloring, wakeup slot	Slot assignment	High	Moderate	High	High
ArDez	Rendezvous-based scheme	Rendezvous period	High	Good	Moderate	Low
DMAC	Staggered wakeup, data prediction	Data-gathering tree	Low	Rather poor	Rather poor	High
CSMA-STDMA	CSMA initializing, STDMA data	Frequent slot assignment	Rather high	Poor	Moderate	High
E-hybrid	Contention period, reserved slots	Slot assignment	Rather high	Poor	Low	High
WirelessHART	Time-synchronized TDMA/CSMA	Slot assignment	High	Poor	Low	High
Z-MAC	LPL, adaptability to contention level	Slot assignment	Low	Poor	Moderate	Moderate
TCS	Token cycle scheduling	Slot scheduling	Moderate	Moderate	Low	High
MMAC-HR	Multichannel, hopping reservation	Hopping reservation	High	Poor	Low	Low
DSP	Multichannel, fast and slow hopping	Multiple rendezvous	High	Poor	Low	Low

**Table 3:** Simulation parameters of clustering performance.

Description	Symbol	Value
Energy consumption of short distance transmission	*ε_fs_*	10 pJ/bit/m^2^
Energy consumption of long distance transmission	*ε_mp_*	0.0013 pJ/bit/m^4^
Energy consumption for digital coding, modulation, filtering, and spreading of the signal	*E_elec_*	50 nJ/bit
Energy for data aggregation	*E_DA_*	5 nJ/bit/signal
Energy for sound-sensing and calculating	*E_sc-sou_*	50 pJ/bit
Energy for picture-sensing and calculating	*E_sc-pic_*	5 nJ/bit
Energy for seismicity-sensing and calculating	*E_sc-sei_*	3 nJ/bit

## Data Availability

Data are available on request.
